# Down’s Syndrome and Triple Negative Breast Cancer: A Rare Occurrence of Distinctive Clinical Relationship

**DOI:** 10.3390/ijms18061218

**Published:** 2017-06-07

**Authors:** Nandini Dey, Amy Krie, Jessica Klein, Kirstin Williams, Amanda McMillan, Rachel Elsey, Yuliang Sun, Casey Williams, Pradip De, Brian Leyland-Jones

**Affiliations:** 1 Center for Precision Oncology, Department of Molecular and Experimental Medicine, Avera Cancer Institute, Sioux Falls, SD 57105, USA; nandini.dey@avera.org (N.D.); Amy.Krie@avera.org (A.K.); Jessica.Klein@avera.org (J.K.); Kirstin.Williams@avera.org (K.W.); Amanda.McMillan@avera.org (A.M.); Rachel.Elsey@avera.org (R.E.); Yuliang.Sun@avera.org (Y.S.); Casey.Williams@avera.org (C.W.); Pradip.De@avera.org (P.D.); 2 Departmental of Internal Medicine, Sanford School of Medicine, University of South Dakota, Sioux Falls, SD 57105, USA

**Keywords:** Down’s syndrome, triple negative breast cancer, tumor suppressor genes, PI3K-mTOR pathway

## Abstract

Down’s syndrome (DS), the most common genetic cause of significant intellectual disability in children and adults is caused by the trisomy of either all or a part of human chromosome 21 (HSA21). Patients with DS mostly suffer from characteristic tumor types. Although individual patients of DS are at a higher risk for acute leukemia and testicular cancers, other types of solid tumors including breast cancers are mostly uncommon and have significantly lower-than-expected age-adjusted incidence rates. Except for an increased risk of retinoblastomas, and lymphomas, the risk of developing solid tumors has been found to be lower in both children and adults, and breast cancer was found to be almost absent (Hasle H., The Lancet Oncology, 2001). A study conducted in the United States found only one death when 11.65 were expected (Scholl T et al., Dev Med Child Neurol. 1982). A recent study examined mammogram reports of women with DS treated in the largest medical facility specifically serving adults with DS in the United States. It was found that only 0.7% women with DS had been diagnosed with breast cancers (Chicoine B et al., Intellect Dev Disabil. 2015). Here we describe a case of breast cancer in a 25-year-old patient with DS. The disease was presented as lymph node positive carcinoma with alterations of tumor suppressor genes characteristic to the triple negative breast cancer subtype. Comprehensive Genomic Profiling (CGP) revealed a wild-type status for *BRCA1*. The CGP report showed a frameshift mutation, A359fs*10 of the tumor suppressor gene *INPP4B* and another frameshift mutation, R282fs*63 of tumor suppressor gene *TP53* in the tumor biopsy as characteristically found in triple-negative breast cancers. The VUS (Variance of Unknown Significance) alteration(s) were identified in *ASXL1* (L1395V), *NTRK1* (G18E), *DDR2* (I159T), *RUNX1* (amplification), *ERG* (amplification), *SOX2* (T26A), *FAM123B* (G1031D), and *HNF1A* (A301T). Bonafide cancer-related genes of chromosome 21 amplified in the patient’s tumor are *RUNX1* and *ERG* genes. After the completion of the radiation, the patient was placed on everolimus which was based on the result of her CGP report. Thus, post-mastectomy radiation therapy was completed with a recommendation for everolimus for one year. During the time of writing of this report, no metastatic lesions were identified. The patient currently has no evidence of disease.

## 1. Background

Down’s syndrome (DS) is a hereditary disease of chromosomal rearrangement(s). Patients with DS are known to exhibit rearrangement(s) of chromosomal material between chromosome 21 and another chromosome. As a result of this rearrangement, a patient bears the usual two copies of chromosome 21, plus extra material from chromosome 21 attached to another chromosome (which take part in the rearrangement). However, the rearrangement or translocation trisomy 21 is not common in the people with Down’s syndrome. More than 90% of Down’s syndrome is caused by an extra copy of chromosome 21, also called trisomy 21.

Triplication of the human chromosome 21 in DS is associated with an altered genetic dosage of different genes including transcription factors. Since appropriate expressions, as well as the functioning of transcription factors, are essential for regulation of different signals within cells, an altered gene dosage becomes crucial for the development of complex diseases including cancers. Patterns of development of various organ-type cancers in DS are unique and emphasize the relationship between chromosome 21 and tumorigenesis. Children with DS have a ~10- to 20-fold higher risk of developing acute lymphoblastic leukemia (ALL) and acute myeloid leukemia (AML), as compared with non-DS children [1]. Certain solid tumors including lymphomas, retinoblastomas, and testicular germ cell tumors, have been reported in individuals with DS [2,3,4]. However, children with DS do not have a uniformly increased risk of developing solid tumors. A decade ago, Patja et al. reported that in a cohort of a little over 1000 women with DS there was no observed case of breast cancers [5]. A national epidemiological study on mortality over 24 years in France had been conducted by Daniel Sage and colleagues. The outcome of the study showed only five deaths from breast cancers in women with DS (68.98 expected (Fisher test; *p* < 0.00005)) [6]. A similar type of study conducted in the United States found comparable results; only one death when 11.65 were expected [7]. A recent study examined mammogram reports of women with DS treated in the largest medical facility specifically serving adults with DS in the United States. The study examined mammogram reports of women with DS. Records of 684 women and results of 993 mammograms were reviewed, including 902 screening and 93 diagnostic mammograms. The study found that only 0.7% women with DS had been diagnosed with breast cancer [8]. In line with the above reports, Martel-Billard et al. studied trisomy 21 and breast cancer and concluded that chromosomal abnormality of DS protects against breast cancers via genetic mechanisms [9]. The development pattern of cancer in DS patients provides a distinct model to improve our understanding of the genetic basis of tumorigenesis as well as mechanisms of sensitivity to anti-cancer drugs. Here, we describe the first case of triple negative breast cancer in a 25-year-old female patient with Down’s syndrome.

## 2. Case Description

**Figure 1:**
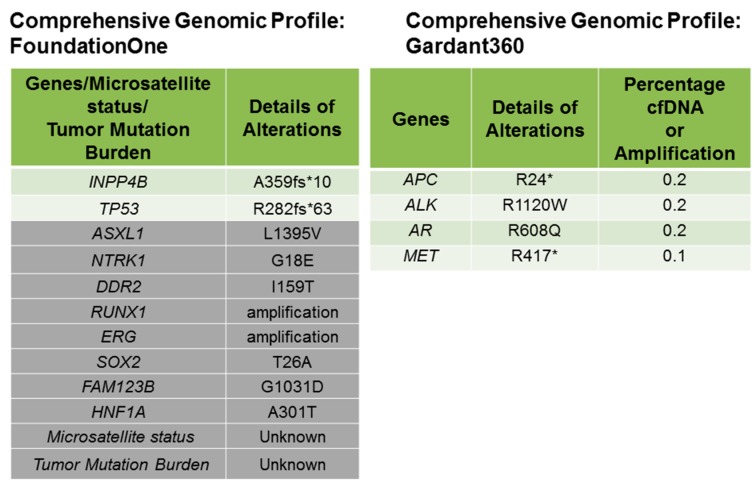
Comprehensive genomic profile of the patient from FoundationOne and Guardant360 data is presented. Genomic alterations identified from FoundationOne data showed a frameshift mutation of *INPP4B* and *TP53*. VUS (variance of unknown significance) are shown in rows in gray. The Guardant360 Table shows the mutant allele percentage (% cfDNA) of observed somatic variants at the basal level (sample submission time point). The “Somatic Alteration Burden” value below refers to the maximum % cfDNA detected at the basal time point. The Guardant360 presents a summary of somatic alterations. The percentage of altered cell-free DNA (% cfDNA) circulating in the blood is measured. The table annotates the allele frequency of altered circulating cell-free DNA (% cfDNA) detected in the patient. Alterations are listed in descending order of % cfDNA by the gene.

**Figure 2:**
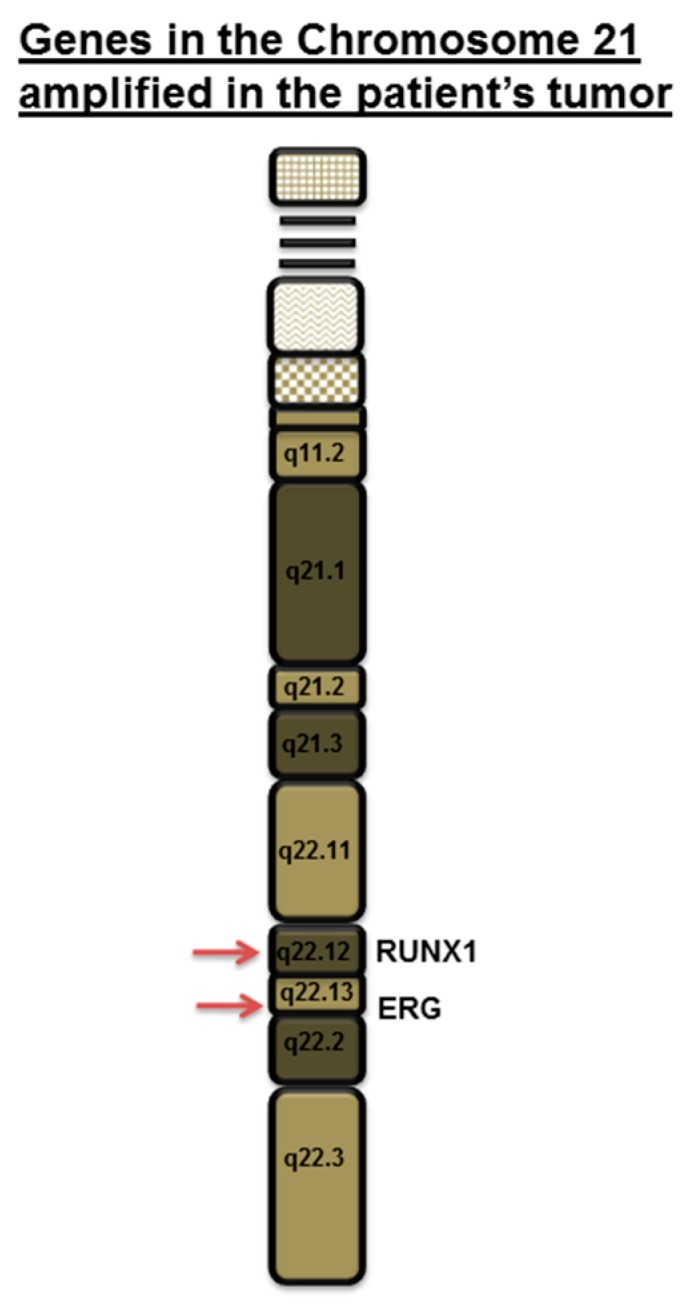
Bonafide cancer-related genes of chromosome 21 amplified in the patient’s tumor. *VUS* genes (Figure 1: in gray shade) from the FoundationOne results show that *RUNX1* and *ERG* genes are amplified in the patient’s tumor.

**Figure 3:**
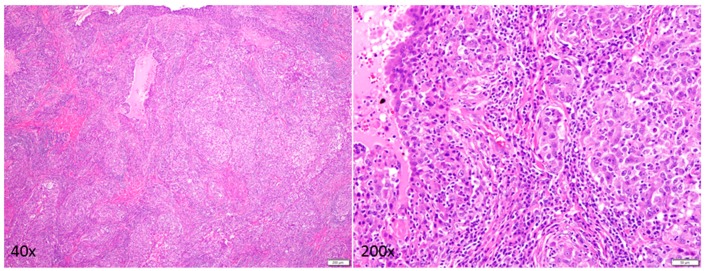
Photomicrograph of H&E stained invasive breast carcinoma (magnification ×40 and magnification ×200): The tumor is composed of large irregular to rounded nests of cells with pleomorphic nuclei and hyperchromasia. Mitoses and dense lymphoplasmacytic infiltration are observed.

**Figure 4:**
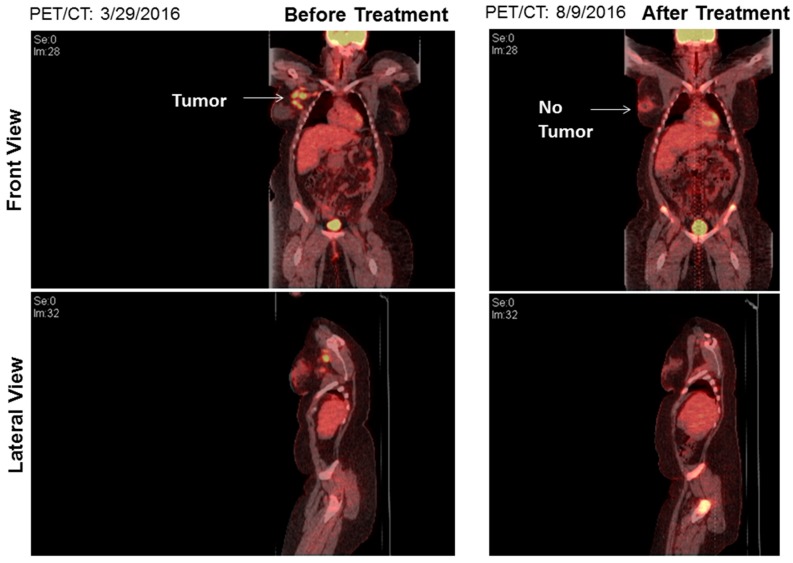
PET/CT of the tumor before (**left** panel) and after (**right** panel) the treatment. The **right** panel shows no evidence of tumor.

A 25-year-old female with DS residing in South Dakota, USA had been found to have a firm, palpable lump in her right upper outer breast 9–10 o′clock position during a physical examination. Patient’s DS has been reported to be mild (otherwise unknown). Patient’s mother’s age was 26 years, and her father’s age was 34 years at the time of the conception. Patient’s mother is a smoker. The patient experienced menarche at the age of 14 years. No major malformations have been reported in the patient. The patient’s paternal aunt was diagnosed with breast cancer in her 70s. No other family history of cancer has been recorded. A diagnostic mammogram was performed, and a mass of 5.4 × 6.1 × 6.2 cm size was identified. The tumor type was diagnosed as breast carcinoma with 5% ER positivity, 0% PR positivity and negative for HER2 (IHC, ratio, and copy number). Ratio and copy numbers are non-detected. Comprehensive Genomic Profiling (CGP) revealed a wild-type status for *BRCA1*. The CGP report showed a frameshift mutation, A359fs*10 of the tumor suppressor gene *INPP4B* and another frameshift mutation, R282fs*63 of tumor suppressor gene *TP53* in the tumor biopsy as characteristically found in triple-negative breast cancers (Figure 1). The VUS (Variance of Unknown Significance) alteration(s) was identified in *ASXL1* (L1395V), *NTRK1* (G18E), *DDR2* (I159T), *RUNX1* (amplification), *ERG* (amplification), *SOX2* (T26A), *FAM123B* (G1031D), and *HNF1A* (A301T). Bonafide cancer-related genes of chromosome 21 amplified in the patient’s tumor have been presented in the Figure 2 which shows that *RUNX1* and *ERG* genes are amplified in the tumor (*VUS* genes in gray shade from the FoundationOne; Figure 1). FoundationOne^®^ provided us fully informative genomic profiles that are based on assays identify the molecular growth drivers of a patient’s cancer (www.foundationone.com). FoundationOne provides validated comprehensive genomic profiles and interrogates the entire coding sequence of 315 cancer-related genes plus select introns from 28 genes those are often rearranged or altered in solid tumors to a typical median depth of coverage of greater than 500×. Each covered read represents a unique DNA fragment to enable the highly sensitive and specific detection of genomic alterations that occur at low frequencies as a result of tumor heterogeneity, limited tumor purity, and a small amount of samples. FoundationOne detects genomic alterations, including base substitutions, insertions and deletions (indels), copy number alterations (CNAs), and rearrangements. The patient received carboplatin plus gemcitabine, and then the treatment was switched to taxol plus carboplatin due to an increase in her liver function test score (LFTS). Patient’s Positron emission tomography/computed tomography (PET/CT) showed an absence of adenopathy. The patient underwent a mastectomy. Right breast mastectomy and axillary lymph node dissection (ALND) showed no residual disease in the breast, but 2 out of 10 lymph nodes (2/10 LN) were found positive. The tumor was Stage III cT3N3M0 Breast Invasive Ductal Carcinoma (IDC) (Figure 3). We tested cfDNA from liquid biopsy by Guardant360. Guardant360 provides the most validated comprehensive liquid biopsy for the 73-gene panel. The digital sequencing technology of Guardant360 identifies the signal of patient’s individual genomic alterations from the noise of interfering data that is inherent to standard next-generation sequencing (NGS) techniques (www.guardanthealth.com). The cfDNA from the liquid biopsy by Guardant360 identified alterations in *APC* (R24*), *ALK* (R1120W), *AR* (R608Q), and *MET* (R417*) indicating a somatic mutation burden of 0.2%. The patient was treated with four cycles of anthracyclin (AC). We followed the Clinical Guidelines for the use of AC × 4 doses. Every two week dosing of doxorubicin and cyclophosphamide for four doses, or dose-dense AC, followed by paclitaxel therapy is listed as a preferred regimen for neoadjuvant and adjuvant treatment in HER-2 negative disease in the National Comprehensive Cancer Network (NCCN) Guidelines for Invasive Breast Cancer Version 2.2017 (NCCN Guidelines. Invasive Breast Cancer. Version 2.2017). In HER-2 positive disease, dose-dense AC followed by trastuzumab, pertuzumab, and paclitaxel or docetaxel is also considered to be a preferred regimen by the NCCN. Every three weeks is considered a lower category of recommendation for these same indications. While it is no longer a preferred regimen for recurrent or metastatic breast cancer, AC is still listed as a treatment option. The American Society of Clinical Oncology (ASCO) also considers the use of an anthracycline-taxane regimen to be the optimal strategy for adjuvant therapy of HER-2 negative or HER-2 positive breast cancers, especially in high risk disease (Selection of Optimal Adjuvant Chemotherapy Regimens for Human Epidermal Growth Factor Receptor 2 (HER2)—Negative and Adjuvant Targeted Therapy for HER2-Positive Breast Cancers: An American Society of Clinical Oncology Guideline Adaptation of the Cancer Care Ontario Clinical Practice Guideline. Journal of Clinical Oncology. 2016). The patient started at outside facility on gemcitabine 1000 mg/m^2^ and carboplatin AUC2 D 1, 8 every 21 days and received two cycles until it was stopped due to persistent aspartate aminotransferase/alanine aminotransferase (AST/ALT) elevation and delayed nausea and vomiting. At that time, she was switched to paclitaxel 80 mg/m^2^ and carboplatin AUC2 D1, 8 q21 days. The patient received four cycles of this regimen for a total of six cycles of chemotherapy. Then she went to surgery, and the pathology report showed that surgical margins were negative and there was no residual cancer in the breast because the patient had previously done an excisional biopsy, but 2 of 10 axillary lymph nodes were positive for metastatic carcinoma. After surgery had been completed, the patient’s family requested a second opinion and the patient started receiving care at Avera Cancer Institute. The risks of cardiac damage in Down’s syndrome was discussed with the family and also increased the risk of leukemia which could be increased by AC treatment for breast cancer but was considered to be beneficial overall considering her high risk of breast cancer relapse. She had a normal multiple-gated acquisition (MUGA) scan and was started on four cycles of AC in the adjuvant setting at a reduced dose of doxorubicin 50 mg/m^2^ and cyclophosphamide 500 mg/m^2^ due to Down’s syndrome. She received radiation to the right breast. After reviewing the pathology further, the ER staining was 5%, and the case was reviewed by the oncotype group and given a prior history of the clot, tamoxifen was decided to be not a right option. After the completion of the radiation, the patient was placed on everolimus which was based on the result of her CGP report. Thus, post-mastectomy radiation therapy was completed with a recommendation for everolimus for one year. The patient currently has no evidence of disease (Figure 4).

## 3. Discussion

One of the privileges of working in cancer clinic is the chance to meet people from all walks of life, many of whom we never get a chance to know otherwise. We report a rare and unexpected co-occurrence of DS and triple negative breast cancer. We seek to find a link between the diseases keeping in mind their state of chromosomal rearrangements and genomic alterations. It is well documented that patients with DS have HSA21 chromosomal rearrangements in different degrees. Korbel et al., mapped HSA21 chromosomal rearrangements across 30 patients in an attempt to interrogate HSA21 at increasingly high resolution [10]. The map revealed (1) subtle translocations and (2) internal rearrangements leading to duplications and deletions of HSA21 regions. For several patients, additional copies of numerous non-contiguous regions were identified by the tiling arrays. It was reported that 30 individuals either had subtle translocations (Dup21JG, Dup21SOS, Dup21DS, Dup21JSB, Dup21NA, Dup21NO, and Dup21BA) or internal rearrangements, duplications, and deletions of HSA21 regions (Dup21JL, Dup21GY, Dup21IS, Dup21JS, Dup21KG, Dup21GP, Dup21KJ, Dup21BS, Dup21HOU, Dup21WA, Dup21SOL, Dup21SM, Dup21ZSC, and Dup21WB) [10]. Collectively, the map revealed complex rearrangements involving multiple events. In a recent study, bursts of chromosome changes have been identified to drive triple negative breast cancers [11]. The study put forward a proposal that complex genomic rearrangements in triple-negative breast cancer cells occur through a “Big Bang” model—early, short bursts of copy number aberrations, rather than progressive accumulation over time. It was postulated that these aberrations are stably maintained in a handful of clones afterward that over time grows to form an active and clinically viable tumor mass. The “Big Bang” model of tumor growth proposes that cancer grows predominantly as a single expansion populated by numerous numbers of intermixed clones. The term was used because this model implies that a signature of the early origins of the malignancy can be theoretically recovered from the present day tumor, in the same way, that the cosmic microwave background represents a signature of the birth of our universe. This implies that both public (clonal) and most private (subclonal) alterations have a distinct connection (The Darwin Cancer Blog: Keeping an evolutionary eye on cancer; https//thedarwincancerblog.com).

**Figure 5:**
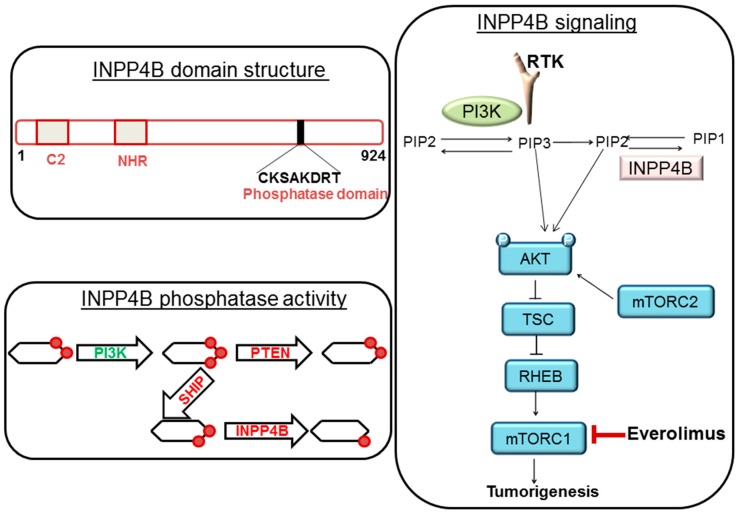
Domain structure, function, and signaling of INPP4B.

In our case, we observed that the tumor is of triple negative subtype. Interestingly, her comprehensive genomic profiling also showed that the tumor contains mutations of both tumor suppressor genes, *TP53* and *INPP4B*. Both *PTEN* and *INPP4B* are often inactivated following mutations in BC, more specifically in the Triple negative breast cancers (TNBC) subtype [12,13]. A substantial number of basal/TNBC patients had an inactivating mutation or loss of INPP4B in both TCGA data set (~30%) [14] and in our Avera TNBC patient cohort. The mechanism of activation of the PI3K-AKT-mTOR pathway in basal-like BC is largely contributed by the frequent genomic alterations of tumor suppressor genes, *PTEN* and *INPP4B* phosphatases [15]. Fedele et al. reported that INPP4B protein expression was frequently lost in primary human breast carcinomas. These primary human breast carcinomas were associated with high clinical grades, tumor sizes, loss of hormone receptors, and were lost most commonly in basal-like breast carcinomas. Their study demonstrated that INPP4B protein loss was also frequently co-existed in PTEN-null tumors. Interestingly, a loss of INPP4B protein was found to be a marker of the aggressiveness in basal-like breast carcinomas [16,17]. From the view point of cell signaling, PTEN and INPP4B control the homeostasis of the activation of the PI3K-AKT signaling pathway. PTEN dephosphorylates PI (3, 4, 5) P3 to PI (4, 5) P2 while INPP4B converts PI (3, 4) P2 to PI (3) P (Figure 5). PI (4, 5) P2 and PI (3) P fail to bind to PH domain of AKT and hence fail to activate AKT which eventually restrict the growth of tumors [18,19]. The PI3K-AKT-mTOR pathway is one of the most commonly activated signaling pathways in human breast cancers. A number of small molecule inhibitors that target various nodes in the pathway have been clinically investigated. Among these agents, first generation allosteric mTOR inhibitor, everolimus has been approved for clinical uses including breast cancer [18]. We also attempted to find the status of genes those are located on the chromosome 21 in the FoundationOne report of the patient. Among many genes present on the chromosome 21 [20], RUNX1 is particularly important as VUS of our patient’s tumor showed an amplification of RUNX1 (Figure 1 and Figure 2). The transcription factor RUNX1 received considerable attention in hematopoietic malignancies [21,22]. RUNX1 was originally identified as a gene fusion in acute myeloid leukemia (AML) and is regarded to play a critical role in the earliest steps of hematopoiesis [21,22]. Importantly, RUNX1 is often expressed in breast epithelium and is deregulated during tumorigenesis. Nicola Ferrari and the team used tissue microarray data of primary breast tumors to study how RUNX1 protein expression is associated with cancer-specific survival in patients with breast cancers [23]. Their study demonstrated that a high expression of the RUNX1 protein was significantly associated with poorer cancer-specific survival in patients with breast cancers belonging to the ER-negative (*p* < 0.05) as well as triple-negative invasive subtypes (*p* < 0.05). RUNX1 alters chromatin structure in cooperation with chromatin modifier and remodeling enzymes and functions both as an oncogene and a tumor suppressor in breast cancers. Barutcu et al., recently reported that RUNX1-mediated perturbation of higher-order genome organization is functionally linked with compromised gene expression in breast cancer cells [24]. Chimge et al., recently reported the relationship between RUNX1 and AXIN1 in ER-negative versus ER-positive breast cancers. RUNX1 plays a tumor suppressor role in estrogen receptor-positive (ER+) disease while it plays an oncogenic role in ER-negative (ER-) tumors. Their study reported that RUNX1 and AXIN1 proteins are strongly correlated in ER- tumors. In their study, the unexpected correlation between RUNX1, playing an oncogenic role in ER- breast cancer, and AXIN1, a well-established tumor suppressor hub, may be related to a high ratio between the expression of variant 2 and variant 1 (v2/v1) of AXIN1 in ER- compared with ER+ breast cancer. The higher v2/v1 ratio in ER- the disease is expected to weaken the tumor suppressor activity of AXIN1 in these tumors [25]. Our study from different breast cancer cohorts has established transcriptional Wnt signaling as a hallmark of triple negative breast cancers [26,27] and the Wnt pathway has been found to be associated with specific metastatic signals [28,29]. It can be argued that an amplification of RUNX1 in our case may have contributed to triggering the Wnt pathway in the context of the genesis of a triple negative breast cancer. Considering the role of RUNX1 in the relapse and metastasis of a tumor as discussed above, we will closely monitor the disease of our patient in the future since her VUS showed amplification of RUNX1.

## 4. Conclusions

To the best of our knowledge, here we describe the first case of triple negative breast cancer in a 25-year-old patient with DS. The disease was presented as lymph node positive carcinoma with alterations of tumor suppressor genes, *TP53* and *INPP4B*, characteristic to the triple negative breast cancer subtype. During the time of writing of this report, no metastatic lesions were identified in the patient, and the PET/CT image did not show any evidence of disease.
